# The Higher Inherent Therapeutic Potential of Biomaterial-Based hDPSCs and hEnSCs for Pancreas Diseases

**DOI:** 10.3389/fbioe.2020.00636

**Published:** 2020-06-26

**Authors:** Bingbing Xu, Fu-Zhen Yuan, Lin Lin, Jing Ye, Bao-Shi Fan, Ji-Ying Zhang, Meng Yang, Dong Jiang, Wen-Bo Jiang, Xing Wang, Jia-Kuo Yu

**Affiliations:** ^1^Knee Surgery Department of the Institute of Sports Medicine, Peking University Third Hospital, Beijing, China; ^2^School of Clinical Medicine, Weifang Medical University, Weifang, China; ^3^Clinical Translational R&D Center of 3D Printing Technology, Shanghai Ninth People's Hospital, Shanghai Jiao Tong University School of Medicine, Shanghai, China; ^4^Beijing National Laboratory for Molecular Sciences, State Key Laboratory of Polymer Physics & Chemistry, Institute of Chemistry, Chinese Academy of Sciences, Beijing, China; ^5^University of Chinese Academy of Sciences, Beijing, China

**Keywords:** hEnSCs, hDPSCs, hADSCs, biomaterial, orthotopic transplantation

## Abstract

Human endometrial stem cells (hEnSCs), dental pulp stem cells (hDPSCs) and adipose tissue-derived stem cells (hADSCs) are considered to be the promising candidates for the treatment of pancreas diseases. The prognosis is better with *in situ* injection of mesenchymal stem cells (MSCs) to the damaged pancreas compared with intravenous injection. However, the clinical application of these cells are limited, due to poor engraftment of transplanted cells after delivery. On the other hand, understanding the role of the biomaterials in cell therapy is essential to promote the therapeutic effects of MSCs. Matrigel, a basement membrane matrix biomaterial, is rich in laminin and collagen IV. The aim of this study is to investigate the difference of biological characteristics of hEnSCs, hDPSCs and hADSCs *in vitro* and their survival situation with Matrigel post intrapancreatic transplantation *in vivo*. Our findings showed, firstly, there was no significant difference in morphology and immunophenotype of these MSCs. Secondly, the biological properties, including cell proliferation, the ability of adipogenic and osteogenic differentiation and the mRNA expression levels of pancreas development-related genes, have been showed distinct difference among these MSCs. Thirdly, Matrigel can improve the survival of MSCs *in vivo*, especially for Matrigel-based hDPSCs and Matrigel-based hEnSCs in pancreas parenchyma of SD rats. These results suggest that hDPSCs and hEnSCs are with the greater inherent therapeutic potential for pancreas diseases compared with hADSCs.

## Introduction

Type 1 diabetes, characterized by insulin deficiency caused by autoimmune destruction of pancreatic beta-cells (American Diabetes Association, 2013), results in an enormous burden on global health and economy. An elegant solution to reintroduce functional insulin-secreting cells in patients is to derive islets, β-cells or pancreatic progenitor cells *in vitro* from human pluripotent stem cells (Pagliuca et al., 2014), which would provide an alternative cell source for cell transplantation therapy in diabetes. Of particular interest is the application of autologous mesenchymal stem cells (MSCs) with the low risk of tumorigenesis (Domínguez-Bendala et al., 2012), no immune rejection and little ethical concerns. Among them, human endometria, dental pulp and adipose tissue–derived MSCs are considered to be the attractive sources for potential clinical application, because of their easy accessibility, high clonogenicity and minimal economic burden and discomfort for donors (Prianishnikov, 1978; Gronthos et al., 2000; Zuk et al., 2002).

The endometrium is highly dynamic and experienced 400 menstrual cycles during a woman's lifetime. In 1978, Prianishnikov first observed that stem cells were present in the endometrium (Prianishnikov, 1978). Human endometrial stem cells (hEnSCs) have specific stem or progenitor cells that have long been believed to be critical for cyclic growth and regeneration (Gargett and Masuda, 2010). Human dental pulp stem cells (hDPSCs) are located in the pulp tissue in the central cavity of the tooth, and are particularly interesting because although tooth is small but still a source of abundant cells for clinical applications. These cells were firstly reported in 2000 by Gronthos and characterized with high clonogenicity, regenerative capacity, and the ability to generate densely calcified nodules (Gronthos et al., 2000). Human adipose tissue-derived stem cells (hADSCs) were firstly isolated from subcutaneous adipose tissue and introduced as a multipotent, undifferentiated and self-renewing cell population by Zuk et al. (2002). ADSCs can be acquired easily from adipose tissue through a minimally invasive method, and the quality and proliferation of ADSCs do not decline with the donor's age (Beane et al., 2014).

Previous studies have demonstrated that MSCs derived from various tissue sources are different in gene expression profile, growth pattern and propensity toward specific lineage (Nekanti et al., 2010). Therefore, it is reasonable that various MSCs producing different cytokines and growth factors that might be more suitable for specific clinical applications. Similarly, we assumed that gene expressions determining the cells development pathway are different among MSCs derived from various sources. Moriscot et al. found that native human bone marrow MSCs constitutively express NKX6.1 at a low level but lack all other transcription factors implicated in β-cell differentiation (Moriscot et al., 2005). In addition, umbilical cord blood contains a subpopulation of cells that are very similar in phenotype to endocrine cell precursors in transition to β-cells (Pessina et al., 2004), but the related gene expression of hEnSCs, hDPSCs and hADSCs in β-cell differentiation remains poorly understood.

A pattern of orderly activation and extinction of many genes during development control the formation of the pancreas and their subsequent differentiation into mature cell types including different exocrine and endocrine cell types. Expression of these related genes is regulated by a hierarchy of key transcription factors, such as SRY-box 17 (Sox17), forkhead box A2 (Foxa2), C-X-C motif chemokine receptor 4 (Cxcr4), pancreatic and duodenal homeobox 1(Pdx1), neuronal differentiation 1 (Neurod1), neurogenin 3 (Ngn3), paired box 4 (Pax4), which control the embryonic formation of pancreatic islets (Jensen, 2004).

On the other hand, the direct injection of MSCs into the damaged pancreas may improve the therapeutic effects compared with intravenous injection. However, these cells are not always function efficiently after injection, due to poor engraftment of transplanted cells, which prevents its wider application. Biomaterials have experienced steady and strong growth over its history and attracted increasing attention for tissue restoration and regeneration (Zhu et al., 2019; Xu et al., 2020b). However, the role of the biomaterials in cell therapy remains unclear. Matrigel is a biomaterial basement membrane matrix, which is derived from mouse sarcoma, and is mainly composed of laminin and collagen IV.

**Figure 1:**
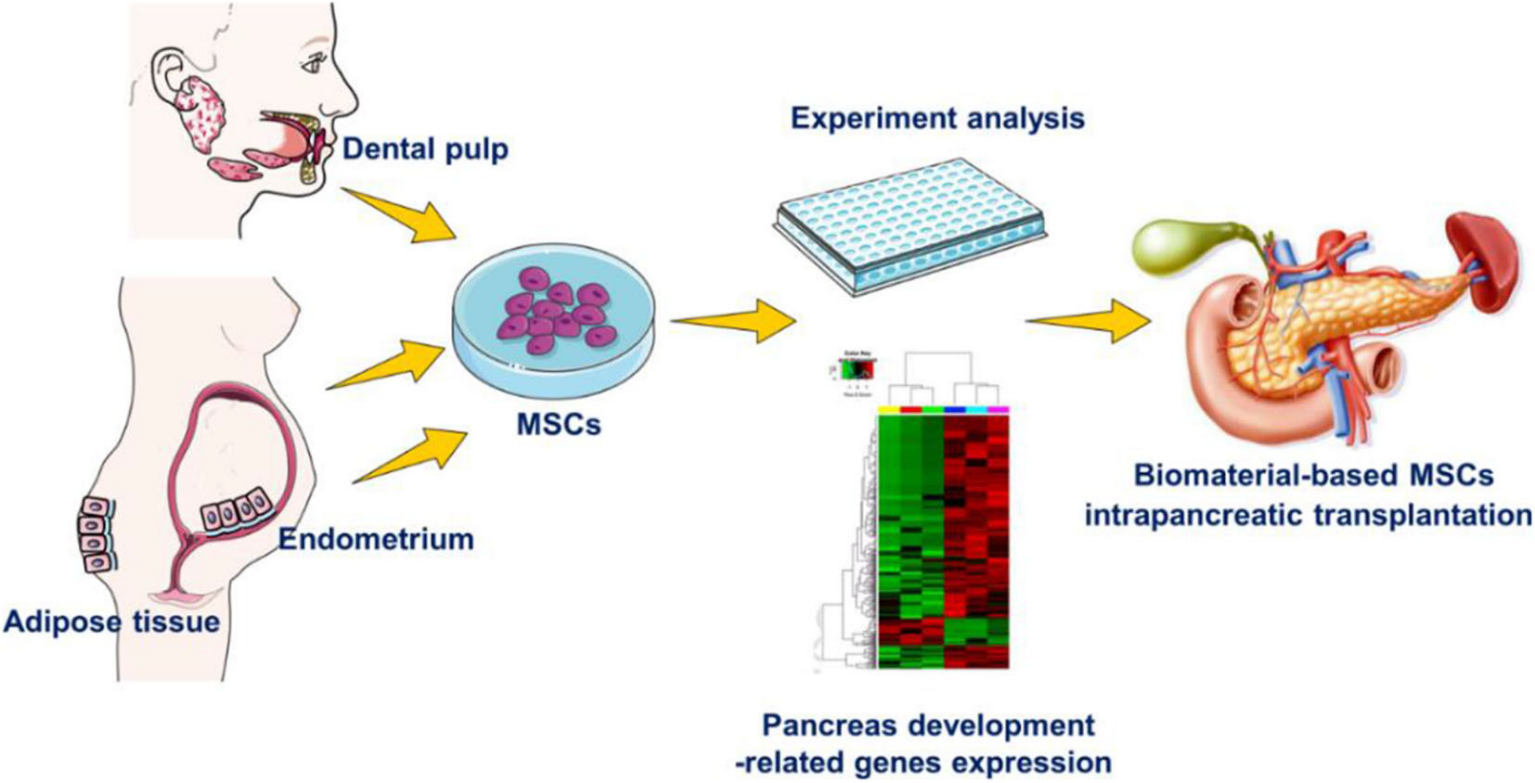
Experimental design. The differences in hEnSCs, hDPSCs and hADSCs in the aspects of biological characteristics *in vitro* and their survival situation with Matrigel biomaterial post intrapancreatic transplantation *in vivo*.

To better understand the inherent therapeutic potential for pancreas diseases of hEnSCs, hDPSCs and hADSCs and the role of biomaterials post intrapancreatic transplantation *in vivo*, first we compared and confirmed that these cells exhibit typical MSC morphology, have high proliferation and multipotency, and express the phenotypic surface marker characteristics of MSCs. Subsequently, we compared the different expression levels of key transcription factors implicated in pancreatic development and function in MSCs by reverse transcription-quantitative polymerase chain reaction (RT-qPCR). Finally, our initial experiments show that the three types of Matrigel-based MSCs can survive differently under pancreatic microenvironment conditions after local injection into pancreatic parenchyma in living SD rats (Figure 1).

## Materials and Methods

### Materials

Dulbecco's modified Eagle's medium/nutrient mixture F-12 (DMEM-F12), penicillin/streptomycin and fetal bovine serum (FBS) were obtained from Gibco. Collagenase P were obtained from Roche. Anti-human CD34-PE, CD45-FITC, HLA-DR-FITC, CD44-FITC, CD73-PE, CD90-FITC, and were purchased from BD Biosciences. Adipogenic and osteogenic inducing medium from Cyagen. Matrigel were obtained from Corning. Trizol Reagent were purchased by Invitrogen. All-In-One RT MasterMix Kit, EvaGreen qPCR MasterMix Kit were obtained from abmGood. Cell-tracker dye CM-DiI were purchased from Invitrogen.

### Isolation and Culture of hEnSCs, hDPSCs and hADSCs

Endometrium tissues were collected from menstrual blood of 5 donors (ages 24–30 year olds females). Adipose tissues were obtained from 5 donors (ages 24–30 year olds females) through liposuction procedures. Intact deciduous tooth were obtained from 5 children (aged 8–12 years) who were undergoing continuous occlusal treatment with tooth extraction. All the donors and guardians provided written informed consents and experiments involving human tissue were approved by Peking University Third Hospital.

The endometrial sliced tissue was digested with 0.1% (w/v) collagenase P for 30 min and followed by the shaking with DNase I (15 U/mL) for another 30 min (37°C, 180 rpm), finally kept in DMEM-F12, adding with 1% (v/v) penicillin/ streptomycin and 10% (v/v) FBS. Nonadherent cells were removed 48 h after initial plating by intensely washing the flasks.

The dental pulp was expose, chopped into small pieces, minced, and digested in a 0.04% (w/v) collagenase solution. Culture conditions and medium were the same as hEnSCs.

The adipose tissue was washed and centrifuged. The adipose tissue in the middle was left and washed. The next procedures, culture conditions and medium were the same as hDPSCs.

### Growth Characteristics Analysis

To compare the growth characteristics of the MSCs at P4, the growth rate and population doubling time (PDT) were measured. The cells in 6-well plates were counted for 7 consecutive days to measure the growth rate by digesting 3 wells and counting the cells. For PDT measure, the cells were counted until they reached 100% confluency. The PDT was calculated using the following formula: PDT = (CT × ln2)/ln(Nf/Ni), where CT is the cell culture time, Ni is the initial number of cells, and Nf is the final number of cells (Chen et al., 2015).

### Flow Cytometry Analysis

For phenotypic identification of the MSCs at P4, cells were stained with the following antibodies (1: 200) for 15 min at room temperature: anti-human CD44-FITC, CD73-PE, CD90-FITC, CD34-PE, CD45-FITC, and HLA-DR-FITC. No antibody is added to the negative control group. The MSCs were washed twice with PBS, resuspended and analyzed by flow cytometry.

### *In vitro* Differentiation Assay

The MSCs at P4 were induced to differentiate into adipocytes and osteoblasts. Briefly, hADSCs were exposed to adipogenic induc medium for 7 days, and hDPSCs and hEnSCs were exposed for 28 days. Then, all MSCs were induced in osteogenic induction medium for 28 days. MSCs were stained with oil red O and alizarin red for adipogenic and osteogenic differentiation evaluation, respectly.

### RNA Isolation and RT-qPCR Analysis

Total cellular RNA of MSCs at P4 was extracted with Trizol Reagent. cDNA was synthesized from RNA by using All-In-One RT MasterMix Kit and qPCR assay was performed with gene specific primers and EvaGreen qPCR MasterMix kit. The primer sequences of qPCR were shown in Table 1.

**Table 1:** Primers sequences used in quantitative polymerase chain reaction.

**Gene**	**Sequence (5^**′**^ to 3^**′**^)**	**Product length (bp)**
*Sox17*	F: GGCGCAGCAGAATCCAGA R: CCACGACTTGCCCAGCAT	61
*Foxa2*	F: AAGACCTACAGGCGCAGCTA R: CCTTCAGGAAACAGTCGTTGA	214
*Cxcr4*	F: AACTGAGAAGCATGACGGACAAGTAC R: GCTGTAGAG GTTGACTGTGTAGATGAG	164
*Pdx1*	F: TGATACTGGATTGGCGTTGT R: GAATGGCTTTATGGCAGATTA	191
*Ngn3*	F: GGCTGTGGGTGCTAAGGGTA R: CAGGGAGAAGCAGAAGGAACAA	104
*Neurod1*	F: GACGACCTCGAAGCCATGAACG R: CCTCCTCTTCCTCTTCTTCCTCCTC	106
*Pax4*	F: GTATGGCTTGGAATGAGGCAGGAG R: GCAATCACAGGAAGGAGGAAGGAG	125
*Insulin*	F: CAGCCGCAGCCTTTGTGA R: GTGTAGAAGAAGCCTCGTTCC	91
*GAPDH*	F: CAGGAGGCATTGCTGATGAT R: GAAGGCTGGGGCTCATTT	138

### *In vivo* Studies of MSCs

The MSCs (about 1 × 10^6^ cells) were labeled with membrane-bound cell-tracker dye CM-DiI (Invitrogen). Matrigel physical embedded and not embedded MSCs were orthotopically transplanted into pancreatic parenchyma of SD rats (*n* = 5 in each group) by local injection, respectively. The survival of the SD rats in each group were observed. The fasting blood glucose levels of the tail tip were monitored at 30 min and day 1 post-implantation and followed by every three other days using a standard blood glucose meter. SD rats were sacrificed 14 days post transplantation, the pancreatic tissues were harvested for cryo-section. The slides with nuclear staining (DAPI) were evaluated to examine the survival of MSCs under pancreatic microenvironment conditions. All animal experiments were approved by Peking University Third Hospital.

### Statistical Analysis

The statistical data are analyzed by GraphPad Prism 5.0 (California, USA). The data are presented as the means ± standard deviation (SD). Bilateral unpaired *t*-test is performed for comparisons between the two groups.

## Results and Discussion

### Morphology and Multilineage Differentiation Potential

**Figure 2:**
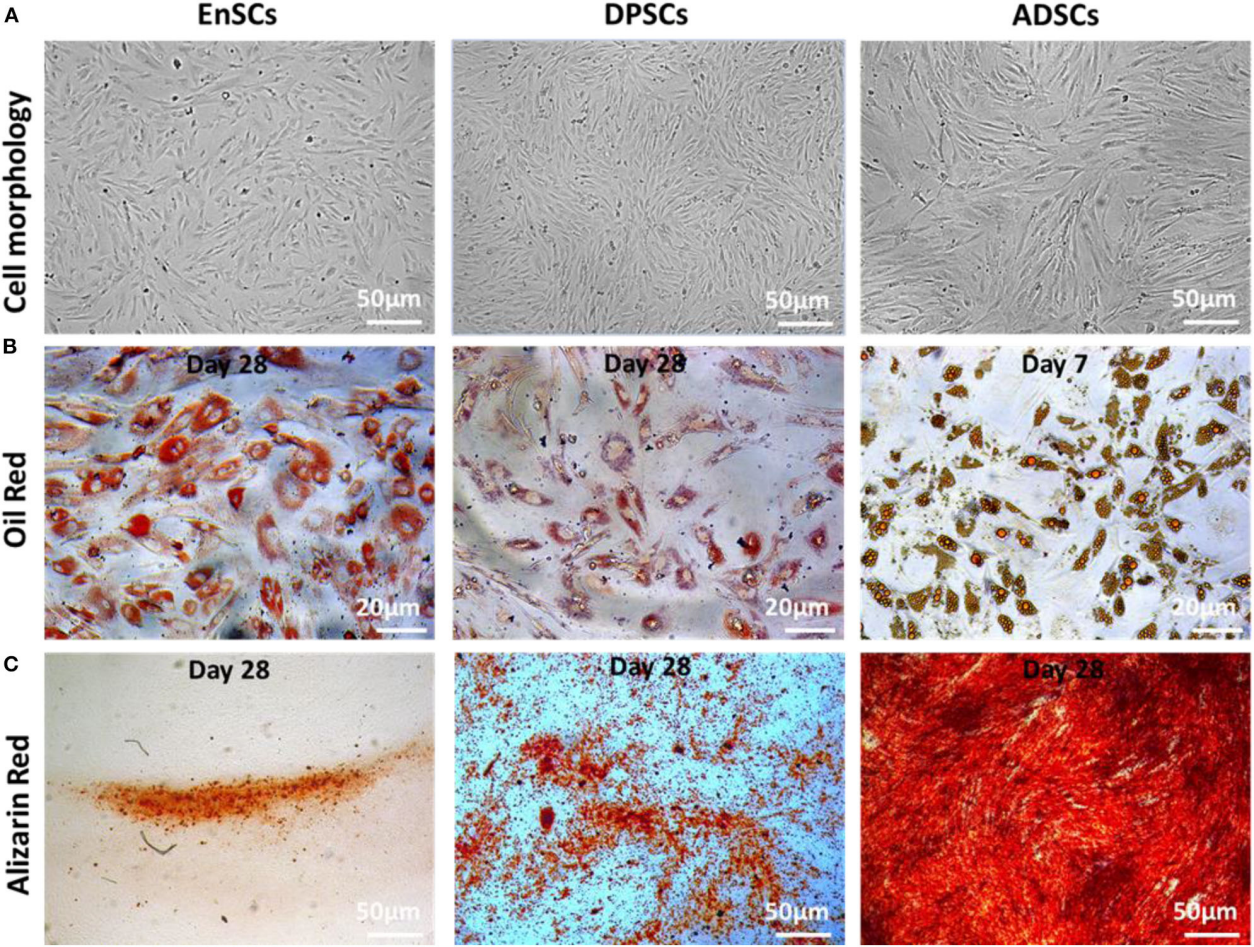
Morphology and multilineage differentiation of MSCs derived from different tissues. All MSCs exhibited spindle-shaped morphology (**A**, scale bar = 50 μm). The differentiation of adipocytes and osteoblasts were verified by Oil Red O (**B**, scale bar = 20 μm) and Alizarin Red (**C**, scale bar = 50 μm), respectively. The representative picture is captured from one of 3 independent experiments.

We found no significant differences in the morphology of three MSCs. All MSCs exhibited typical MSC morphology, including fibroblast-like, spindle shape (Figure 2, top panel). To examine the differentiation potential of the MSCs, the cells at P4 were induced to differentiate into the osteogenic and adipogenic lineages (Figure 2). Lipid droplets began to appear in hADSCs, hEnSCs and hDPSCs on about day 2, 16, and 21, respectively. The accumulation of cytoplasmic lipid vacuoles was obvious in the hADSCs on day 7, whereas only very small lipid granules were detected in hEnSCs and hDPSCs on day 28 and the adipogenic differentiation of hDPSCs were lower than hEnSCs (Figure 2B). In addition, hADSCs exhibited significant osteogenic phenotypes, and the osteogenic differentiation in hEnSCs were only a little positive (Figure 2C).

Our results suggested that hEnSCs and hDPSCs show delayed adipogenic and osteogenic differentiation compared with hADSCs; the adipogenic differentiation of hDPSCs and the osteogenic differentiation of hEnSCs was lower than other MSCs in this study. Our findings, for the first time, revealed that hEnSCs had greater adipogenic competence. hADSCs showed significant osteogenic phenotypes in previous study (Zuk et al., 2002), and our data further demonstrated that hADSCs exhibited higher osteogenic differentiation compared with hEnSCs and hDPSCs. Additionally, hDPSCs showed more remarkable osteogenic potential compared with hEnSCs as well, which was consistent with the previous publication (Tabatabaei and Torshabi, 2017).

### Immunophenotype

**Figure 3:**
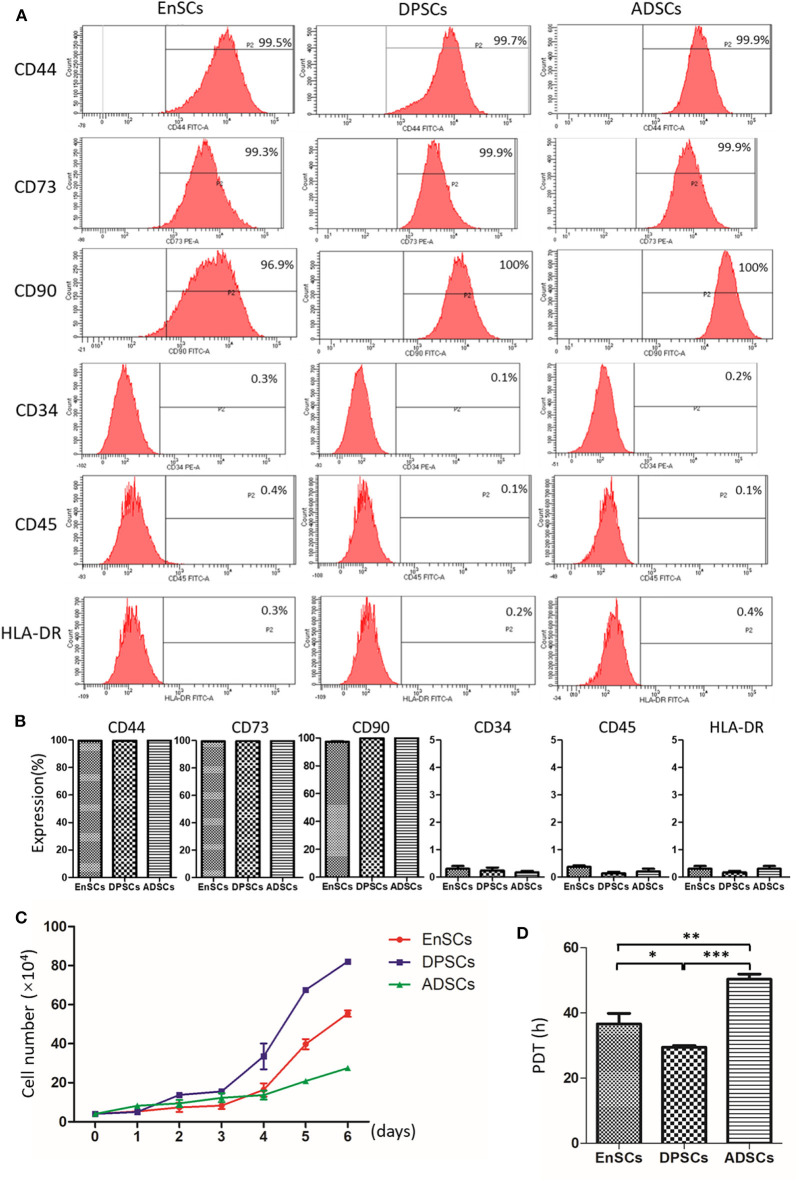
Immunophenotype and proliferative potential of hEnSCs, hDPSCs and hADSCs. **(A,B)** Flow cytometric analysis of the expression of surface markers on MSCs. Shown **(A)** is one representative of 3 independent experiments. **(C)** Growth curves of MSCs (*n* = 3 donors). **(D)** PDT of MSCs from different types were analyzed and compared (*p < 0.05, **p < 0.01, ***p < 0.0001).

The International Society for Cellular Therapy (ISCT) criteria (Dominici et al., 2006) defined the classical MSCs phenotypic markers. All of these MSCs were highly expressed with MSC-specific surface markers (CD44, CD73, and CD90), and lowly expressed with hematopoietic cell marker (CD34), leucocyte marker (CD45), and monocyte/macrophage marker (HLA-DR). Our results showed there was no significant difference of immunophenotype in the MSCs (*n* = 3) (Figures 3A,B).

### Growth Characteristics of MSCs

The growth curves of hEnSCs, hDPSCs and hADSCs at P4 show that the proliferation ability of the MSCs was hDPSCs > hEnSCs > hADSCs (Figure 3C). Furthmore, the cell PDT of the hEnSCs, hDPSCs and hADSCs were 36.62 ± 3.19 h, 29.45 ± 0.54 h, 50.34 ± 1.56 h, respectively (Figure 3D). The previous study showed the proliferation rate of hEnSCs were greater than that of hDPSCs from adult teeth (Tabatabaei and Torshabi, 2017), which was not contradictory with our results because hDPSCs from deciduous teeth in our study showed higher proliferation rate than hDPSCs from adult teeth (Wang et al., 2018). Meanwhile, hDPSCs from adult teeth were highly proliferative compared with hADSCs (Abu Kasim et al., 2015), which was consistent with our results.

### Gene Expression Analysis

**Figure 4:**
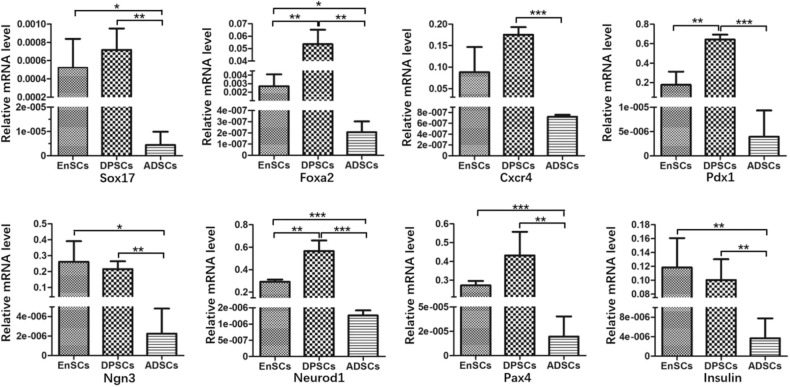
Gene expression analysis of hEnSCs, hDPSCs and hADSCs. The expression levels relative to the housekeeping gene (GAPDH) are shown. The data are presented as the means ± standard deviation (SD) of 3 experiments; *p < 0.05, **p < 0.01, ***p < 0.0001.

Relative mRNA expression levels of pancreas development-related genes in MSCs were analyzed using RT-qPCR (Figure 4). The results showed that hEnSCs, hDPSCs and hADSCs all expressed these genes. The mRNA expression levels of *Sox17, Foxa2, Ngn3, Neurod1, Pax4*, and *Insulin* from hADSCs were markedly lower than hEnSCs and hDPSCs. High expressions of *Foxa2, Pdx1*, and *Neurod1* were observed in hDPSCs compared with hEnSCs, but there were no significant differences in the expression of *Sox17, Cxcr4, Ngn3, Pax4*, and *Insulin* among these MSCs. Interestingly, the mRNA expressions of *Foxa2, Pdx1*, and *Neurod1* in hDPSCs were at least twenty times, three times, twice as high as in hEnSCs, respectively. hADSCs expressed significantly lower level of *Cxcr4* and *Pdx1* than hDPSCs, and no significant difference in the expression of *Cxcr4* was observed compared with hEnSCs.

In our study, MSCs all naturally express *Sox17, Foxa2, Cxcr4, Pdx1, Neurod1, Ngn3, Pax4*, and *Insulin*, and the mRNA expression levels of these genes showed great variation. Govindasamy et al. revealed that the gene variations of the stem cells determine the lineage propensity toward a specific destination (Govindasamy et al., 2010). It has been demonstrated that MSCs from dental pulp and adipose tissue can differentiate into functional insulin-producing cells by using the same three-step protocol, but the yield of PDX-1^+^ cells and C-peptide-positive cells from hDPSCs were 44.92 ± 0.9% and 30.56 ± 2%, respectively; and that from murine epididymal ADSCs were 57–71% and 47–51%, respectively (Chandra et al., 2010; Govindasamy et al., 2011). The different yield of PDX-1^+^ cells and C-peptide-positive cells may be related to the inherent expressions of pancreas development-related genes.

In previous studies, researchers succeeded in obtaining islet-like cell aggregates using a stepwise method, from MSCs to endoderm-like cells, pancreatic progenitor-like cells, and β cells (Chandra et al., 2010; Govindasamy et al., 2011; Li et al., 2014). *Sox17, Foxa2*, and *Cxcr4*, three relatively specific marker genes for definitive endoderm (Li et al., 2014). We found that the expression of *Sox17* in the hADSCs was obviously lower compared with the other two kinds of MSCs and there were no significant differences between hDPSCs and hEnSCs. *Sox17* drives human pluripotent stem cells toward an endodermal or mesendodermal fate (Guo and Hebrok, 2009). For another two endodermal markers, hDPSCs expressed the highest level of *Foxa2* but expression of Cxcr4 only higher than hADSCs. hADSCs expressed significantly less *Foxa2* than hEnSCs. *Foxa2* is essential for the cell type–specific transcription of the *Pdx1* gene in the differentiation of the pancreas (Lee et al., 2002). *Cxcr4* is essential for pancreatic endocrine progenitor cells proliferation and maturation (Guo and Hebrok, 2009). Based on these results, it is possible that the potential of MSCs reprograming into definitive endoderm-like cells was as follows: hDPSCs > hEnSCs > hADSCs.

*Pdx1*, one specific marker gene for pancreatic progenitors; *Ngn3* and *Neurod1*, two endocrine progenitor marker genes (Li et al., 2014). *Pdx1* is the only transcription factor specific of the endocrine pathway (Moriscot et al., 2005). *Ngn3* is crucial for the induction of differentiation from pancreatic progenitor cells into endocrine cells and switches off before the final differentiation into β cells (Watada, 2004). *Neurod1* plays a crucial role in endocrine cell survival and *Insulin* gene transcription (Watada, 2004). In this study, strong expressions of *Pdx1* and Neurod1 were observed in hDPSCs compared to that of hEnSCs. The expression levels of *Ngn3* and *Neurod1* in hADSCs were the lowest in the three MSCs, but the expression of *Pdx1* only less than hEnSCs. These results were surprising in that *Pdx1* could control *Ngn3* in activating the expression of other differentiation factors for endocrine cells, while *Neurod1* is activated by *Ngn3* (Pessina et al., 2004; Watada, 2004). Furthermore, it is of possibility as well that the potential of MSCs from definitive endoderm-like cells into pancreatic progenitors-like cells was as follows: hDPSCs > hEnSCs > hADSCs.

*Pax4* is essential to initiate pancreatic cell differentiation (Guo and Hebrok, 2009). Furthermore, we observed that expression levels of *Pax4 and Insulin* in hADSCs were the lowest as compared with hEnSCs and hDPSCs, while no significant differences in the expressions of *Pax4* and *Insulin* were observed in hDPSCs compared to that of hEnSCs. Most importantly, the *Insulin* mRNA is expressed in all the MSCs, but undifferentiated hEnSCs, hDPSCs and hADSCs can't release *Insulin* in response to glucose *in vitro* (Chandra et al., 2010; Govindasamy et al., 2011; Santamaria et al., 2011). All the mRNA expression levels of these pancreas development-related genes suggest that potential post-transcriptional regulating mechanisms may block the protein expression of some key transcription factors implicated in pancreatic development. And it can be hypothesized that the silent genes reside in the three MSCs in a standby state, and the activation of the genes are restricted by their natural environment.

### The Survival of Matrigel-Based MSCs Post Transplantation and the Effects of Matrigel-Based MSCs to SD Rats

**Figure 5:**
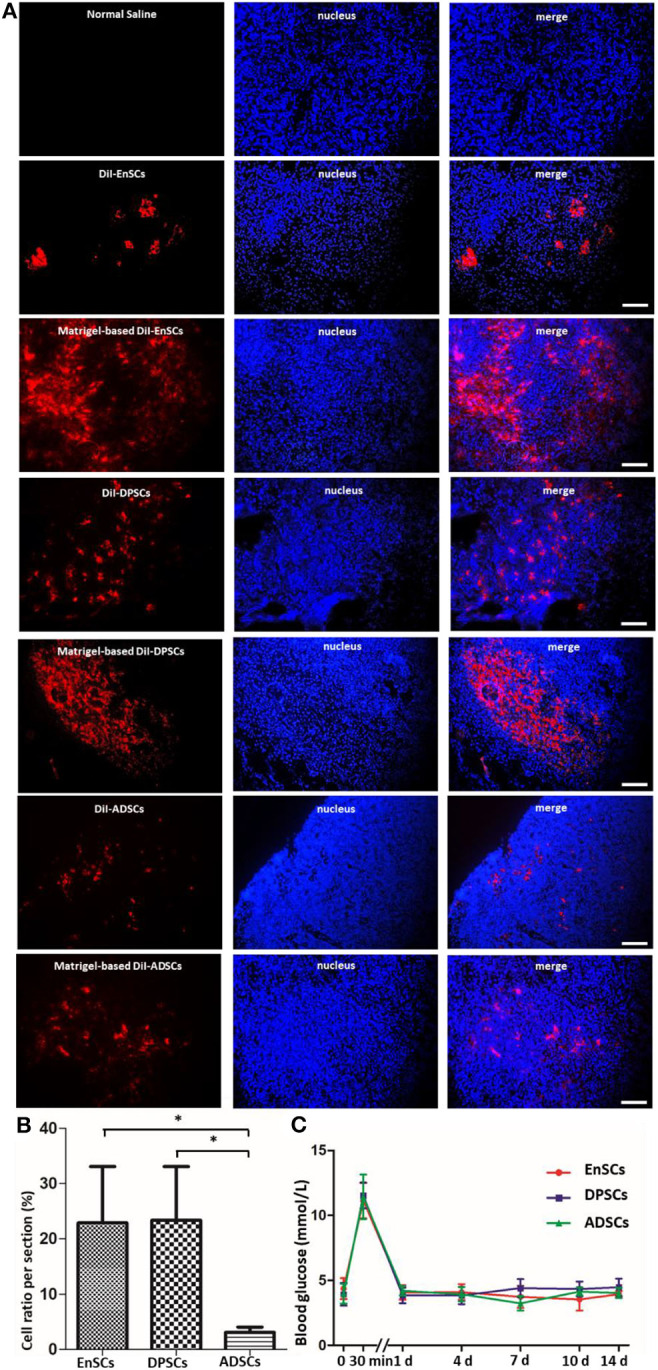
MSCs from three sources at day 14 post orthotopic injection into pancreatic parenchyma of SD rats. **(A)** Representative fluorescence micrographs of cryo-sections in the EnSCs (upper panel), DPSCs (middle panel) and ADSCs (bottom panel) engraftment groups 14 days after orthotopical transplantation into pancreatic parenchyma of SD rats, respectively. DiI fluorescence, red; DAPI fluorescence, blue. Scale bar = 20 μm. **(B)** The average ratios of Matrigel-based CM-DiI-labeled cells counted in the pancreatic cryo-sections. Shown in the histogram were the percentages of Matrigel-based CM-DiI-labeled MSCs in the three groups compared to the total nuclei. *p < 0.05. **(C)** Blood glucose levels in SD rats transplanted orthotopically with Matrigel-based hEnSCs, Matrigel-based hDPSCs, and Matrigel-based hADSCs. Shown are the average blood glucose levels of the tail tip measured and plotted for each time point.

Direct check of cryo-section under a Immunofluorescence microscope showed that all the CM-DiI-labeled MSCs could be detected in the pancreatic tissues on day 14 post-transplanation (Figure 5A). The average ratios of CM-DiI-labeled cells in Matrigel-based hDPSCs (23.4 ± 5.58%) and Matrigel-based hEnSCs (22.88 ± 5.89%) counted per pancreatic section were significantly higher than Matrigel-based hADSCs (3.09 ± 0.57%) (*p* < 0.05) (Figure 5B). These results indicated that Matrigel could improve the survival of MSCs and Matrigel-based hDPSCs, and Matrigel-based hEnSCs had higher survival rates than Matrigel-based hADSCs in pancreas parenchyma of SD rats, which was consistent with the mRNA expression levels of pancreas development-related genes. It is suggested that mRNA expressions of these genes may reflect the survival of MSCs in pancreas.

The blood glucose levels of the rats were increased instantaneously at 30 min after the implantation of Matrigel-based MSCs and then decreased to normal levels at day 1 post-implantation. There were no significant difference among these MSCs (Figure 5C). These results suggested that the pancreatic orthotopic engraftment of MSCs did little damage to the pancreas, and pancreatic orthotopic engraftment of hDPSCs and hEnSCs could be used for clinical treatment.

These data indicate that Matrigel-based hDPSCs, Matrigel-based hEnSCs and Matrigel-based hADSCs could survive in the pancreatic parenchyma of SD rats. Low-temperature Matrigel suspension and injection was effective for establishing an orthotopic mouse model of pancreatic cancer (Jiang et al., 2014). The three-dimensional culture of hDPSCs in Matrigel promotes the differentiation of DPSCs into insulin-producing Cells (Xu et al., 2020a). Matrigel could also be used for the renal subcapsular transplant of native mouse islets and pancreatic progenitor-like cells (Li et al., 2014). However, there is no research on the role of biomaterials in pancreas transplantation of MSCs. Our findings, for the first time, revealed that Matrigel can improve the survival of MSCs, and Matrigel-based hDPSCs and Matrigel-based hEnSCs had higher survival rates than Matrigel-based hADSCs in pancreas.

## Conclusion

In summary, our novel and unexpected finding is a step forward in evaluating the high inherent therapeutic potential of hDPSCs and hEnSCs for pancreas diseases. Nevertheless, further investigations are needed to identify the ability of MSCs derived from the two sources differentiating into insulin-producing β-cells.

## Data Availability Statement

The raw data supporting the conclusions of this article will be made available by the authors, without undue reservation.

## Ethics Statement

The studies involving animal and human participants were reviewed and approved by Peking University Third Hospital. Written informed consent to participate in this study was provided by the participants' legal guardian/next of kin.

## Author Contributions

XW and J-KY conceived the research. BX, F-ZY, JY, J-YZ, MY, and B-SF isolated and cultured the MSCs. J-YZ analyzed the growth characteristics. BX and LL performed the Flow cytometry. BX and B-SF did the RT-qPCR analysis and transplantation experiment. BX wrote the manuscript. DJ and W-BJ helped revising the paper. All authors contributed to the article and approved the submitted version.

## Conflict of Interest

The authors declare that the research was conducted in the absence of any commercial or financial relationships that could be construed as a potential conflict of interest.
